# Association between the C-reactive protein-triglyceride-glucose index and asthma risk: evidence from the NHANES cohort and validation in the CHNS cohort

**DOI:** 10.1016/j.clinsp.2026.100965

**Published:** 2026-04-23

**Authors:** Junmin Qian, Peng Xu, Ziying Peng, Zuoxun Xia

**Affiliations:** a Yongjia People’s Hospital, Wenzhou, Zhejiang, China; b Guizhou Medical University, Guiyang, Guizhou, China; c Department of Pathology, the Third Affiliated Hospital of Nanchang University, Jiangxi Medical College, Nanchang University and the First Hospital of Nanchang City, Nanchang, Jiangxi, China

**Keywords:** C-Reactive Protein-Triglyceride Glucose Index, Asthma, National Health and Nutrition Examination Survey, China Health and Nutrition Survey

## Abstract

**Background:**

Asthma is a chronic respiratory disorder characterized by airway inflammation and reversible airflow limitation. Emerging evidence highlights the roles of systemic inflammation and metabolic dysregulation in its pathogenesis. The C-reactive protein-Triglyceride glucose Index (CTI), a novel composite biomarker integrating inflammatory and metabolic components, may reflect these processes, yet its association with asthma remains unclear.

**Methods:**

Data from 18,579 adults in the National Health and Nutrition Examination Survey (NHANES, 1999–2010, 2015–2018) were analyzed to examine the relationship between CTI and asthma prevalence. Logistic regression models with progressive adjustments for demographic, lifestyle, and clinical covariates were applied. Nonlinear and threshold relationships were evaluated using restricted cubic splines. The authors performed subgroup analyses in participants with metabolically unhealthy obesity and in never-smokers, and conducted a brief association validation using data from the China Health and Nutrition Survey (CHNS) 2009 (*n* = 6220).

**Results:**

In NHANES, each unit increase in CTI was associated with a 20 % higher asthma likelihood after full adjustment (OR = 1.20, 95 % CI: 1.04–1.40, *p* = 0.018). Subgroup analyses indicated that the positive association remained robust among participants with metabolically unhealthy obesity and never-smokers. The association appeared linear, with no evidence of a significant threshold effect. The CHNS cohort confirmed the positive association (OR = 1.61, 95 % CI: 1.11–2.31, *p* = 0.012).

**Conclusions:**

A stable positive association was observed between CTI and asthma, suggesting that CTI may serve as a convenient biomarker for risk assessment; however, longitudinal studies are warranted for further validation.

## Highlights

• CTI correlates with increased asthma risk in NHANES and CHNS cohorts.
• No nonlinear relationship found between CTI and asthma risk.
• CTI integrates inflammation and metabolism for asthma risk assessment.

## Introduction

Asthma represents a prevalent chronic respiratory condition distinguished by airway inflammation, airway hyperresponsiveness, and reversible airflow limitation as its core pathological features.^1^ According to global epidemiological data, approximately 300 million individuals are affected by asthma, and its disease burden continues to rise.^1^^,^^2^ Recent studies have reported that asthma accounted for an estimated 457,010 deaths in 2017.^3^ increasing to approximately 461,100 deaths in 2019.^4^ thereby imposing a substantial burden on global public health systems and resulting in considerable socioeconomic losses.

Localized airway inflammation is considered the pathological core of asthma.^5^ accumulating evidence indicates that asthma patients universally exhibit a state of low-grade systemic inflammation.^6^^,^^7^ Multiple clinical investigations have confirmed that serum high-sensitivity C-Reactive Protein (hs-CRP) levels in individuals with asthma are markedly elevated versus healthy controls, with this elevation being more pronounced in patients with poorly controlled disease.^8^^,^^9^ Furthermore, hs-CRP levels demonstrate a significant positive correlation with asthma severity, suggesting that systemic inflammation serves a crucial function in the pathological advancement of asthma.

Furthermore, recent research has revealed that the pathogenesis of asthma is not limited to immune-inflammatory responses, as metabolic abnormalities also play a pivotal role.^10^^,^^11^ Metabolic dysfunction can exacerbate airway inflammation and asthma symptoms by enhancing inflammatory cascade reactions.^10^ The Triglyceride-Glucose index (TyG), functioning as a dependable surrogate marker for evaluating insulin resistance, exhibits a markedly elevated pattern in asthma patients.^12^, ^13^, ^14^ further substantiating the important role of metabolic factors in asthma pathogenesis.

The pivotal roles of inflammation and metabolic abnormalities in asthma pathogenesis have been widely acknowledged, existing research exhibits significant limitations. Most studies focus solely on the independent effects of individual biomarkers; for instance, some investigations exclusively examine the impact of inflammatory indicators such as hs-CRP on asthma, while others solely explore the roles of metabolic parameters, including body mass index and insulin resistance. However, systematic research on the potential interactions between inflammation and metabolism remains relatively scarce. This limitation in research perspective constrains comprehensive understanding and in-depth insight into the complex pathological mechanisms underlying asthma.

C-reactive protein-Triglyceride glucose Index (CTI) is an emerging composite biomarker that has gained attention in recent years.^15^ It systematically integrates inflammatory markers (C-reactive protein) with metabolic indicators (triglyceride-glucose index), thereby enabling simultaneous assessment of both systemic inflammatory status and the degree of lipid metabolic dysregulation. Current literature has demonstrated that CTI exhibits strong links to diverse chronic conditions, encompassing stroke, diabetes mellitus, and cardiovascular diseases.^16^, ^17^, ^18^ Nevertheless, investigations examining the link between CTI and asthma constitute a predominantly unexplored domain that requires urgent and thorough examination.

Therefore, this investigation seeks to systematically examine the link between CTI and asthma, offering comprehensive insights into the potential function of the inflammation-metabolism axis in asthma development, with the objective of establishing novel theoretical foundations and practical clinical assessment tools for early identification, risk stratification, and formulation of individualized precision intervention strategies for asthma.

## Methods

### Study population

This cross-sectional study used data from the National Health and Nutrition Examination Survey (NHANES) as the primary analytic cohort to investigate the association between the CTI and asthma. To assess the robustness of the findings, the authors conducted prespecified subgroup analyses within the NHANES cohort among participants with metabolically unhealthy obesity and among never-smokers. In addition, data from the China Health and Nutrition Survey (CHNS) were used as an independent external validation cohort to perform a brief replication analysis of the observed association.

NHANES, overseen by the National Center for Health Statistics (NCHS), is a major public health initiative designed to track and assess the health and nutritional well-being of both adults and children in the United States. The dataset provides a wealth of detailed information, covering demographics, dietary habits, clinical exam results, lab tests, survey responses, and restricted-access records. The term “Continuous NHANES” refers to information gathered since 1999, with biennial releases and ongoing updates.

Given the objective constraints related to data availability and research environment, the temporal scope selection of this study necessitates comprehensive consideration of the following key elements: First, during the 2011‒2012 to 2013‒2014 cycles, limitations in data completeness precluded the accurate computation of CTI. Second, in the period from 2019 onwards, the global outbreak of COVID-19 markedly interfered with the physiological status of the target study population.

**Fig. 1:**
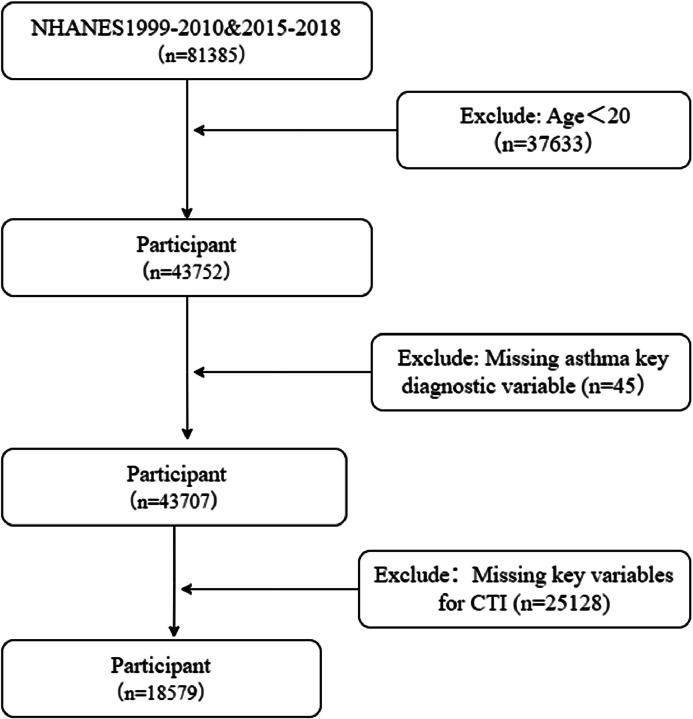
Participant screening flow chart of the NHANES population.

This study finally comprised 81,385 participants from eight NHANES survey cycles, covering the periods from 1999‒2000 to 2009‒2010 and from 2015‒2016 to 2017‒2018. The conditions for inclusion were delineated as follows: Individuals aged 20-years and above who participated in any of the eight designated survey cycles. The exclusion criteria included: 1) Subjects lacking essential characteristics for asthma diagnosis; 2) Participants missing fundamental factors required for CTI calculation. A sum of 18,579 participants who met the study criteria were included in the statistical analysis. Fig. 1 depicts the complete participant selection flowchart. The baseline characteristics of the prescreened NHANES cohort are provided in Supplementary Table 1.

### Calculation of CTI

The CTI is a composite metric used to evaluate individual metabolic health status by integrating CRP, Triglycerides (TG), and glucose levels to capture both inflammatory and metabolic conditions. A growing body of evidence has linked the CTI to a range of chronic diseases, including stroke, diabetes, and cardiovascular disease.^16^, ^17^, ^18^ however, its association with asthma remains to be elucidated.

The CTI was calculated employing the subsequent equation: CTI=0.412×Ln(CRP)(mg/dL)+Ln[triglyceride(mg/dL)×fastingglucose(mg/dL)/2].^19^

### Definition of asthma

Patients who answered “Yes”[20] to the NHANES questionnaire item, “Has a doctor or other health professional ever informed you that you have asthma?” were categorized as possessing asthma.

### Covariates

Covariate information was gathered through standardized survey instruments, encompassing demographic and lifestyle factors encompassing age, sex, ethnicity, educational level, Poverty Income Ratio (PIR), marital status, and total caloric intake on the first day. Smoking classification comprised three groups: never smokers (individuals with fewer than 100 lifetime cigarettes), former smokers (individuals with >100 lifetime cigarettes who discontinued), and current smokers (individuals actively smoking). The authors extracted the continuous variable “amount of alcohol consumed” and categorized alcohol intake according to average daily drinks. Alcohol intake was stratified as light consumption (≤ 1 drink/day for women or ≤ 2 drinks/day for men over the previous 12-months), moderate consumption (1–3 drinks/day for women or 2–4 drinks/day for men), and heavy consumption (≥ 4 drinks/day for women or ≥ 5 drinks/day for men).^20^ Physical activity was categorized into low and high levels.^21^ All subjects filled out a comprehensive physical activity survey that captured every bout of exercise they had engaged in during the past month. The form documented the nature, length, intensity level, and frequency of each activity over the preceding 30-days. Moderate-intensity pursuits were characterized by activities that caused light perspiration or a modest to noticeable uptick in respiration or heart rate. On the other hand, vigorous exercises were those that resulted in heavy sweating or substantial elevations in breathing patterns or heart rate. Specific Metabolic Equivalent (MET) values were assigned to each activity based on its type and intensity level. These MET values were then multiplied by the average duration and frequency of occurrence within the last 30-days to determine the MET minutes per 30-days (MET min/30d) for each individual activity. The MET min/30d figures for all activities were subsequently added together before being divided by 4.29 to compute the total weekly MET minutes. Before conducting the analysis, participants were divided into two groups ‒ low and high physical activity ‒ depending on whether they satisfied the recommended national physical activity benchmarks (low physical activity < 500 MET/wk; high physical activity ≥ 500 MET/wk).

Hypertension was defined as possessing a history of hypertension or recorded Systolic Blood Pressure (SBP) ≥ 140 mmHg or Diastolic Blood Pressure (DBP) ≥ 90 mmHg. The presence of cardiovascular disease was determined based on physician-confirmed diagnoses reported by participants through standardized health questionnaires administered during one-on-one interviews. Participants who responded positively to any of these conditions ‒ congestive heart failure, coronary heart disease, angina pectoris, myocardial infarction, or stroke ‒ were classified as having cardiovascular disease.^22^

According to the guidelines set forth by the National Cholesterol Education Program's Adult Treatment Panel III, metabolic syndrome was diagnosed when an individual exhibited at least three of the specified risk factors. These included having a waist measurement of 102 cm or more for men and 88 cm or more for women (with different thresholds for Asian populations: 90 cm for Asian men and 80 cm for Asian women), elevated triglyceride levels of 150 mg/dL or higher or current use of lipid-lowering drugs, low high-density lipoprotein cholesterol below 40 mg/dL in males or 50 mg/dL in females or taking cholesterol medication, blood pressure readings of 130/85 mm/Hg or greater or antihypertensive drug therapy, and fasting blood glucose levels at or above 100 mg/dL or being on medication for diabetes mellitus.^23^

The authors determined the estimated Glomerular Filtration Rate (eGFR) using the 2021 formula from the Chronic Kidney Disease Epidemiology Collaboration (CKD-EPI).^24^ For research purposes, chronic kidney disease was characterized by either an eGFR below 60 mL/min/1.73 m^2^ or a Urine Albumin-to-Creatinine Ratio (UACR) of 30 mg/g or higher.^25^

NAFLD is often diagnosed using the U.S. Fatty Liver Index (USFLI), which requires a score of 30 or more. This definition is widely accepted and has been shown to be reliable, boasting an area under the receiver operating characteristic curve, or AUROC, of 0.80, with a 95 % Confidence Interval ranging from 0.77 to 0.83, for accurately predicting the presence of NAFLD as confirmed by ultrasound.^26^ Glycated hemoglobin was directly extracted from the NHANES database, and Body Mass Index (BMI) was calculated using measured height and weight.

### Statistical analysis

Statistical examination incorporated sampling weights, cluster analysis, and stratification to account for NHANES’ sophisticated multi-tiered probability framework. Baseline data presented continuous variables as means alongside standard deviations, assessed through autonomous sample *t*-tests when normally distributed. For categorical parameters, frequencies plus percentages were displayed and evaluated using Chi-Square analysis. The link between CTI and asthma underwent examination via binary logistic regression methodology. Three analytical models were constructed in this study. Model 1 was unadjusted. Model 2 was adjusted for demographic and lifestyle-related covariates, including age, sex, ethnicity, marital status, smoking status, educational level, Amount of alcohol consumed, physical activity, PIR, calorie intake on the first day, and BMI. Model 3 was further adjusted for clinical factors based on Model 2, including hypertension, cardiovascular disease, metabolic syndrome, CKD, NAFLD, and glycated hemoglobin.

Smooth curve fitting and threshold effect assessments were executed to explore potential nonlinear associations between CTI and asthma susceptibility. To assess the robustness and external validity of these findings, the authors performed subgroup analyses within the NHANES cohort among participants with metabolically unhealthy obesity and among never-smokers, and further conducted sensitivity analyses using the CHNS cohort. As a nationally representative longitudinal study in China, CHNS has a well-documented database design, quality control, and sample representativeness in peer-reviewed literature; thus, technical details are not repeated here.^27^^,^^28^ The authors analyzed 6220 participants with complete data from the 2009 CHNS, including 89 asthma cases (prevalence 1.4 %). Because asthma events were rare, the authors used Firth’s penalized likelihood logistic regression to mitigate small-sample bias and enhance model stability. Analyses were conducted in *R* (version 4.2.1), and two-sided p-values < 0.05 were considered significant.

## Results

### Baseline demographic characteristics of the NHANES cohort

A total of 18,579 participants were included in this study, of whom 16,162 did not have asthma (86.99 %) and 2417 had asthma (13.01 %). Significant between-group differences were observed in age, marital status, PIR, BMI, CTI, sex, ethnicity, smoking status, hypertension, metabolic syndrome, cardiovascular disease, and NAFLD (all *p* < 0.05). In contrast, no statistically significant differences were found between the two groups with respect to HbA1c, amount of alcohol consumed, calorie intake on day-1, physical activity, educational attainment, alcohol use, or CKD (all *p* > 0.05). Complete baseline characteristics are described in Table 1.

**Table 1:** Baseline characteristics of participants included in the NHANES cohort.

Variables	Total (*n* = 18,579)	Asthma		Statistic	p-value
		No (*n* = 16,162)	Yes (*n* = 2417)		
Glycated hemoglobin (%), mean (SE)	5.56 (0.01)	5.56 (0.01)	5.58 (0.02)	*t* = 1.03	0.303
Age, mean (SE)	47.25 (0.24)	47.54 (0.27)	45.39 (0.40)	*t*=−4.81	**<0.001**
PIR, mean (SE)	3.03 (0.03)	3.05 (0.03)	2.85 (0.06)	*t*=−3.94	**<0.001**
Amount of alcohol consumed (drinks), mean (SE)	2.69 (0.04)	2.69 (0.04)	2.72 (0.09)	*t* = 0.32	0.746
Calorie intake (kcal/d), mean (SE)	2188.30 (10.22)	2185.93 (10.97)	2203.25 (33.90)	*t* = 0.48	0.635
BMI, mean (SE)	28.67 (0.08)	28.47 (0.08)	29.93 (0.22)	*t* = 6.61	**<0.001**
CTI, mean (SE)	7.95 (0.01)	7.93 (0.01)	8.07 (0.03)	*t* = 5.09	**<0.001**
Marital status, n (%)				χ^2^=64.06	**<0.001**
Married	9901 (57.10)	8783 (57.99)	1118 (51.51)		
Widowed	1623 (6.08)	1447 (6.23)	176 (5.11)		
Divorced	1851 (9.89)	1531 (9.45)	320 (12.66)		
Separated	602 (2.50)	508 (2.45)	94 (2.83)		
Never married	2975 (16.59)	2487 (16.06)	488 (19.91)		
Living with partner	1405 (7.83)	1202 (7.81)	203 (7.99)		
Sex, n (%)				χ^2^=61.63	**<0.001**
Male	8928 (48.54)	7936 (49.69)	992 (41.32)		
Female	9651 (51.46)	8226 (50.31)	1425 (58.68)		
Ethnicity, n (%)				χ^2^=67.04	**<0.001**
Mexican American	3617 (8.19)	3370 (8.79)	247 (4.41)		
Other Hispanic	1506 (5.54)	1274 (5.40)	232 (6.40)		
Non-Hispanic White	8466 (68.98)	7278 (68.91)	1188 (69.45)		
Non-Hispanic Black	3622 (10.73)	3057 (10.42)	565 (12.68)		
Other Race-Including Multi-Racial	1368 (6.57)	1183 (6.49)	185 (7.06)		
Smoking status, n (%)				χ²=42.78	**<0.001**
Never	9884 (52.17)	8746 (53.11)	1138 (46.20)		
Former	4810 (26.14)	4131 (25.70)	679 (28.87)		
Now	3866 (21.70)	3269 (21.19)	597 (24.93)		
Education, n (%)				χ^2^=3.22	0.415
Less than high school	5293 (17.94)	4698 (18.13)	595 (16.75)		
Highschool or equivalent	4328 (25.01)	3762 (25.03)	566 (24.92)		
College or above	8929 (57.04)	7675 (56.84)	1254 (58.33)		
Alcohol use, n (%)				χ^2^=7.49	0.085
Mild drinking	5645 (50.38)	4906 (50.75)	739 (48.15)		
Moderate drinking	3603 (33.00)	3067 (32.51)	536 (35.98)		
Heavy drinking	2044 (16.62)	1779 (16.74)	265 (15.87)		
Physical activity, n (%)				χ^2^=4.27	0.106
Low physical activity	5663 (33.43)	4896 (33.11)	767 (35.42)		
High physical activity	9140 (66.57)	7890 (66.89)	1250 (64.58)		
Hypertension, n (%)				χ^2^=13.23	**0.001**
No	10,698 (63.21)	9386 (63.72)	1312 (59.98)		
Yes	7807 (36.79)	6704 (36.28)	1103 (40.02)		
Metabolic syndrome, n (%)				χ^2^=7.25	**0.038**
No	12,854 (74.64)	11,246 (75.00)	1608 (72.41)		
Yes	4443 (25.36)	3798 (25.00)	645 (27.59)		
CKD, n (%)				χ^2^=1.51	0.353
No	15,071 (86.94)	13,113 (87.06)	1958 (86.18)		
Yes	3287 (13.06)	2856 (12.94)	431 (13.82)		
Cardiovascular diseases, n (%)				χ^2^=57.08	**<0.001**
No	16,366 (91.24)	14,357 (91.87)	2009 (87.29)		
Yes	2106 (8.76)	1714 (8.13)	392 (12.71)		
NAFLD, n (%)				χ^2^=35.95	**<0.001**
No	7690 (66.21)	6809 (67.23)	881 (59.59)		
Yes	4279 (33.79)	3657 (32.77)	622 (40.41)		

SE, Standard Error; BMI, Body Mass Index; PIR, Poverty Income Ratio; CTI, C-reactive protein-Triglyceride-glucose Index; NAFLD, Non-Alcoholic Fatty Liver Disease; CKD, Chronic Kidney Disease.

As shown in Table 2, after adjustment for covariates in Model 3, each 1-unit increase in CTI was associated with 20 % higher odds of asthma (OR = 1.20, 95 % CI 1.04–1.40; *p* = 0.018), indicating a positive association between CTI and asthma.

**Table 2:** Association between CTI and asthma.

Variables	Model 1		Model 2		Model 3	
	OR (95 % CI)	p-value	OR (95 % CI)	p-value	OR (95 % CI)	p-value
CTI	1.16 (1.10 ∼ 1.23)	**<0.001**	1.15 (1.05 ∼ 1.25)	**0.004**	1.20 (1.04 ∼ 1.40)	**0.018**

OR, Odds Ratio; CI, Confidence Interval; CTI, C-reactive protein-Triglyceride glucose Index; NAFLD, Non-Alcoholic Fatty Liver Disease; CKD, Chronic Kidney Disease.

Model 1: No adjustment was made for potential confounders.

Model 2: Adjusted for age, PIR, sex, ethnicity, marital status, smoking status, educational level, amount of alcohol consumed, physical activity, BMI, and calorie intake on the first day.

Model 3: Adjusted for age, PIR, sex, ethnicity, marital status, smoking status, educational level, amount of alcohol consumed, physical activity, calorie intake on the first day, BMI, hypertension, cardiovascular disease, metabolic syndrome, CKD, NAFLD, and glycated hemoglobin.

### Exploration of non-linear relationships

**Fig. 2:**
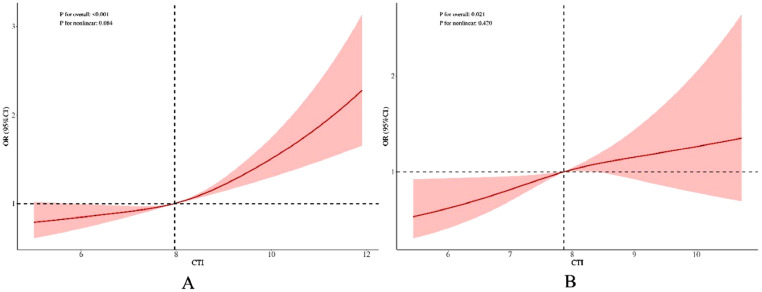
RCS analysis of the association between CTI and asthma risk. (A) RCS analysis of CTI and asthma risk in univariate logistic regression. (B) RCS analysis of CTI and asthma risk in multivariate logistic regression. (B) The model was adjusted for the following covariates: age, sex, ethnicity, marital status, smoking status, educational level, alcohol consumption, physical activity, PIR, first-day calorie intake, BMI, hypertension, cardiovascular disease, metabolic syndrome, CKD, NAFLD, and glycated hemoglobin. OR, Odds Ratio; CI, Confidence Interval; CTI, C-reactive protein-Triglyceride glucose Index; PIR, Poverty Income Ratio; BMI, Body Mass Index; CKD, Chronic Kidney Disease; NAFLD, Nonalcoholic Fatty Liver Disease.

Both univariate and multivariate logistic regression analyses demonstrated a marked link between CTI and asthma risk. Nevertheless, subsequent Restricted Cubic Spline (RCS) analysis failed to detect evidence of a nonlinear relationship between CTI and asthma risk (Fig. 2).

**Fig. 3:**
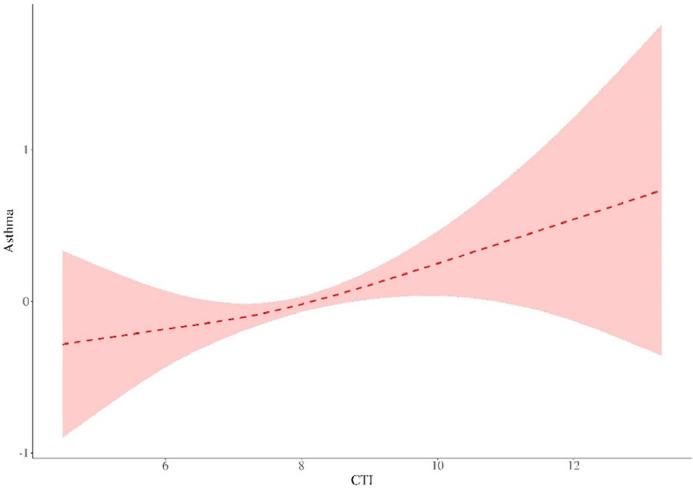
**Threshold effect analysis of the link between CTI and asthma risk.** Threshold effect analysis was performed using the segmented package in R. The model was adjusted for age, sex, ethnicity, marital status, smoking status, educational level, alcohol consumption, physical activity, PIR, first-day calorie intake, BMI, hypertension, cardiovascular disease, metabolic syndrome, CKD, NAFLD, and glycated hemoglobin. CTI, C-reactive protein-Triglyceride glucose Index; PIR, Poverty Income Ratio; BMI, Body Mass Index; CKD, Chronic Kidney Disease; NAFLD, Nonalcoholic Fatty Liver Disease.

To further examine potential nonlinear associations, the authors performed threshold effect analysis (Fig. 3, Table 3), indicating no substantial threshold effect in the link between CTI and asthma risk.

**Table 3:** Threshold effect analysis between CTI and asthma risk.

Outcome	Effect	p-value
Model 1 Fitting model by standard linear regression	1.05 (0.92‒1.20)	0.445
Model 2 Fitting model by two-piecewise linear regression		
Inflection point	8.234	
< 8.234	1.22 (0.97‒1.53)	0.084
≥ 8.234	0.82 (0.58‒1.16)	0.270
P for likelihood test		0.333

### Sensitivity analysis

To strengthen the present conclusions, the authors conducted a subgroup analysis within the NHANES cohort focusing on participants with metabolically unhealthy obesity, a phenotype that has been well documented in prior research.^29^^,^^30^ Obesity was identified based on a BMI threshold of 30.0 kg/m^2^ or higher. To gauge metabolic dysfunction, the authors looked for signs like: 1) SBP of 130 mmHg or more, DBP of 85 mmHg or higher, or reliance on antihypertensive drugs; 2) Fasting plasma glucose of 100 mg/dL or higher, or the use of antidiabetic medication; 3) High-density lipoprotein cholesterol levels below 40 mg/dL for males and below 50 mg/dL for females; or 4) Triglycerides readings of 150 mg/dL or above.

As shown in Supplementary Table 2, the positive association between CTI and asthma remained robust in the metabolically unhealthy obese subgroup.

Additionally, the authors conducted a subgroup analysis among never-smokers (Supplementary Table 3). In the fully adjusted logistic regression model (Model 3), the positive association between CTI and asthma remained robust.

To assess the robustness of the primary findings across different populations, the authors conducted a supplementary association analysis in the CHNS cohort.

As shown in Table 4, among 6220 participants with complete data from the 2009 CHNS cohort, 89 had asthma, yielding a prevalence of 1.4 %.

**Table 4:** Baseline characteristics of 6220 participants in the 2009CHNS cohort.

**Characteristic**	**Asthma**	
	**No (*n* = 6131)**	**Yes (*n* = 89)**
Sex, n (%)		
Male	2948 (48.1)	54 (60.7)
Female	3183 (51.9)	35 (39.3)
Hypertension, n (%)		
No	5203 (84.9)	62 (69.7)
Yes	928 (15.1)	27 (30.3)
Myocardial infarction, n (%)		
No	6063 (98.9)	87 (97.8)
Yes	68 (1.1)	2 (2.2)
Stroke, n (%)		
No	6037 (98.5)	82 (92.1)
Yes	94 (1.5)	7 (7.9)
CTI, mean (SE)	4.03 (0.53)	4.18 (0.50)
Age, mean (SE)	52.39 (14.59)	63.84 (12.31)
BMI, mean (SE)	23.89 (3.51)	24.08 (4.71)

BMI, Body Mass Index; CTI, C-reactive protein-Triglyceride glucose Index.

To examine the link between CTI and asthma risk in the 2009 CHNS cohort, the authors applied Firth’s penalized likelihood logistic regression. Given that only 89 cases of asthma were identified (prevalence 1.4 %), representing a rare-event scenario, including an excessive number of covariates in the model could result in insufficient events per variable, leading to overfitting and overly wide confidence intervals. Although Firth’s method reduces small-sample bias, it cannot fully eliminate instability caused by the limited number of events. Therefore, to ensure robustness and minimize overadjustment bias, the authors employed univariate Firth logistic regression, focusing on estimating the main effect of CTI on asthma risk. The analysis revealed a marked positive link between CTI and asthma (OR = 1.61, 95 % CI 1.11–2.31, *p* = 0.012).

## Discussion

This study systematically explored the association between CTI and asthma risk using data from the NHANES cohort, and further validated the stability of this association across different populations using data from the CHNS cohort. The study found a positive correlation between CTI levels and the risk of asthma development. These results deepen the understanding of the role of the inflammation-metabolism pathway in the pathogenesis of asthma and suggest that CTI may serve as a novel biological marker for asthma risk assessment, potentially providing a new approach for disease prevention and early therapeutic interventions.

Traditionally, asthma has been considered an immune-mediated disease characterized primarily by airway inflammation.^31^ However, accumulating evidence indicates that systemic inflammatory responses and metabolic dysregulation play critical roles in the development and progression of asthma.^10^^,^^32^ Previous epidemiological studies have reported that elevated serum hs-CRP levels are markedly linked to disease severity and poor asthma control.^33^ Moreover, metabolic abnormalities ‒ particularly insulin resistance and obesity ‒ can contribute to persistent airway inflammation through multiple mechanisms, including pro-inflammatory cytokine release, enhanced oxidative stress, and imbalances in adipokine secretion.^34^^,^^35^

By integrating inflammatory markers (CRP) and metabolic indices (TyG), CTI provides a more comprehensive assessment of the systemic state of the inflammation-metabolism axis. The present findings extend the predictive utility of CTI beyond cardiovascular diseases and diabetes, and for the first time, explore its association with asthma risk in large-scale population-based cohorts. This suggests that CTI may represent a promising complementary biomarker for asthma risk assessment and disease management. It should be noted, however, that CTI is proposed as an integrative indicator that captures the interplay between inflammation and metabolic dysregulation, rather than as a replacement for its individual components in all clinical scenarios. In specific clinical contexts where isolated inflammatory or metabolic evaluation is required, CRP and TyG may retain independent diagnostic value, and CTI should be considered as a supplementary tool that offers additional pathophysiological insight into the inflammation-metabolism crosstalk underlying asthma susceptibility.

From a mechanistic perspective, the interplay between inflammation and metabolic disturbances may underlie the observed association between CTI and asthma. CRP, as an acute-phase reactant, can promote the release of inflammatory cytokines, thereby amplifying airway inflammation and hyperresponsiveness.^36^^,^^37^ Meanwhile, lipid metabolic disorders reflected by the TyG index may induce oxidative stress and endothelial dysfunction, further exacerbating systemic inflammation.^38^^,^^39^ Although the pathological hallmark of asthma is localized airway inflammation, systemic inflammation may influence pulmonary function via circulating mediators, leading to more severe reversible airflow limitation.^40^ Notably, the authors did not observe a nonlinear relationship between CTI and asthma risk, suggesting a potential linear trend, in which higher CTI levels may exert a stronger impact on asthma through the inflammation–metabolism axis.

Given that CRP and TyG-related metabolic indices are routinely measured in clinical practice, CTI may function as a practical instrument for prompt detection and risk assessment of asthma. Compared with single biomarkers, CTI integrates multidimensional information, is readily available in routine testing, and may help guide personalized intervention strategies, such as dietary modifications targeting metabolic abnormalities or anti-inflammatory therapies. With the global burden of asthma continuing to increase, the application of CTI may potentially mitigate the public health impact and offer a new avenue for precision medicine.

Nonetheless, certain constraints merit consideration. First, given its cross-sectional design, the research cannot determine cause-and-effect relationships, suggesting the necessity for long-term prospective investigations to validate these observations. Second, asthma ascertainment was based on self-reported physician diagnoses, which may be subject to recall error and diagnostic misclassification. In particular, current and former smokers comprised 53.8 % of the asthma group, potentially increasing diagnostic overlap with Chronic Obstructive Pulmonary Disease (COPD) and Asthma-COPD Overlap (ACO), thereby raising concerns about outcome misclassification. To address this issue, the authors performed a subgroup analysis restricted to never-smokers (*n* = 1138), in which the association between CTI and incident asthma remained consistent, lending support to the robustness of the present findings. However, despite these supportive results, residual misclassification between asthma, COPD, and ACO cannot be fully excluded, as self-reported diagnoses lack the specificity of objective pulmonary function testing (e.g., spirometry with bronchodilator reversibility). Such misclassification may have biased the effect estimates in either direction, and future studies incorporating standardized diagnostic criteria and objective lung function measurements are warranted to further clarify these associations.

Third, CTI was calculated using CRP, triglyceride, and glucose concentrations, which are susceptible to short-term biological variability; future studies incorporating longitudinal or dynamic monitoring may improve measurement precision. In addition, future research should leverage well-phenotyped clinical asthma cohorts with comprehensive characterization ‒ encompassing allergic and non-allergic phenotypes and standardized severity grading ‒ to determine whether CTI exerts differential effects across asthma subtypes and to further elucidate the relationship between CTI and asthma severity.

Fourth, due to limited asthma cases (*n* = 89) in the CHNS cohort, univariable analyses were used instead of multivariable models to avoid overfitting, aiming to verify the direction and reproducibility of associations found in NHANES. However, this approach cannot fully control for confounders, potentially introducing residual confounding bias and weakening the robustness of external validation results.

## Conclusion

This study demonstrates a significant correlation between CTI measurements and asthma susceptibility, supporting the role of inflammatory-metabolic interactions in asthma development. However, the cross-sectional design limits causal inference. Future prospective studies and mechanistic analyses are needed to clarify underlying biological pathways and assess whether interventions targeting CTI components ‒ such as lipid-lowering therapy, glycemic optimization, or anti-inflammatory strategies ‒ could modify asthma risk or slow disease progression. Such research would strengthen the causal link between the inflammation-metabolism axis and asthma pathogenesis, and potentially advance CTI from a risk stratification biomarker to a therapeutic monitoring tool, enhancing its clinical utility in asthma prevention and management.

## Ethics approval and consent to participate

The NHANES and CHNS datasets are publicly available and fully de-identified; therefore, no supplementary ethical clearance was essential for this investigation.

## Consent for publication

Not applicable.

## Data availability

The research data utilized in this study are available by contacting the corresponding author directly via a formal request.

## Funding

This investigation did not receive any specific grant from funding agencies in the public, commercial, or not-for-profit sectors.

## Declaration of competing interest

The authors declare no conflicts of interest.

## Appendix Supplementary materials

Image, application 1
