# Supplementary material for: Association between the C-reactive protein-triglyceride-glucose index and asthma risk: evidence from the NHANES cohort and validation in the CHNS cohort
Source: Clinics (Sao Paulo). 2026 Apr 23;81:100965. doi: 10.1016/j.clinsp.2026.100965 (PMC13126462; doi:10.1016/j.clinsp.2026.100965)
Supplement: Supplementary file 1 [file mmc1.docx]

**Supplementary Table 1.Baseline characteristics of the pre-screening and eligible NHANES cohorts**

| Variables | Pre-screening cohort | | | | Eligible cohort | | | |
| --- | --- | --- | --- | --- | --- | --- | --- | --- |
|  | Total (n = 77582) | Asthma | | *P-value* | Total (n = 18579) | Asthma | | *P-value* |
|  |  | No (n=66795) | Yes(n=10787) |  |  | No(n=16162) | Yes(n=2417) |  |
| Glycated hemoglobin. (%), Mean (SE) | 5.51 (0.01) | 5.51 (0.01) | 5.52 (0.01) | 0.617 | 5.56 (0.01) | 5.56 (0.01) | 5.58 (0.02) | 0.303 |
| Age, Mean (SE) | 37.15 (0.19) | 37.47 (0.21) | 35.22 (0.32) | **<0.001** | 47.25 (0.24) | 47.54 (0.27) | 45.39 (0.40) | **<0.001** |
| PIR, Mean (SE) | 2.87 (0.03) | 2.90 (0.03) | 2.71 (0.04) | **<0.001** | 3.03 (0.03) | 3.05 (0.03) | 2.85 (0.06) | **<0.001** |
| Amount of alcohol consumed (drinks), Mean (SE) | 2.75 (0.03) | 2.76 (0.04) | 2.68 (0.06) | 0.182 | 2.69 (0.04) | 2.69 (0.04) | 2.72 (0.09) | 0.746 |
| Calorie intake (kcal/d),Mean(SE) | 2134.08 (6.33) | 2129.56 (6.32) | 2161.46 (17.67) | 0.076 | 2188.30 (10.22) | 2185.93 (10.97) | 2203.25 (33.90) | 0.635 |
| BMI, Mean (SE) | 26.60 (0.06) | 26.44 (0.06) | 27.57 (0.15) | **<0.001** | 28.67 (0.08) | 28.47 (0.08) | 29.93 (0.22) | **<0.001** |
| CTI, Mean (SE) | 7.83 (0.01) | 7.82 (0.01) | 7.89 (0.02) | **0.004** | 7.95 (0.01) | 7.93 (0.01) | 8.07 (0.03) | **<0.001** |
| Marital status, n(%) |  |  |  | **<0.001** |  |  |  | **<0.001** |
| Married | 22908 (53.11) | 20345 (54.09) | 2563 (46.98) |  | 9901 (57.10) | 8783 (57.99) | 1118 (51.51) |  |
| Widowed | 4137 (6.00) | 3678 (6.13) | 459 (5.22) |  | 1623 (6.08) | 1447 (6.23) | 176 (5.11) |  |
| Divorced | 4362 (9.34) | 3651 (9.03) | 711 (11.27) |  | 1851 (9.89) | 1531 (9.45) | 320 (12.66) |  |
| Separated | 1451 (2.41) | 1226 (2.37) | 225 (2.71) |  | 602 (2.50) | 508 (2.45) | 94 (2.83) |  |
| Never married | 13958 (21.97) | 11694 (21.27) | 2264 (26.33) |  | 2975 (16.59) | 2487 (16.06) | 488 (19.91) |  |
| Living with partner | 3326 (7.18) | 2852 (7.13) | 474 (7.49) |  | 1405 (7.83) | 1202 (7.81) | 203 (7.99) |  |
| Sex, n(%) |  |  |  | **<0.001** |  |  |  | **<0.001** |
| Male | 38081 (48.82) | 32851 (49.37) | 5230 (45.45) |  | 8928 (48.54) | 7936 (49.69) | 992 (41.32) |  |
| Female | 39501 (51.18) | 33944 (50.63) | 5557 (54.55) |  | 9651 (51.46) | 8226 (50.31) | 1425 (58.68) |  |
| Ethnicity, n(%) |  |  |  | **<0.001** |  |  |  | **<0.001** |
| Mexican American | 18069 (9.63) | 16397 (10.20) | 1672 (6.19) |  | 3617 (8.19) | 3370 (8.79) | 247 (4.41) |  |
| Other Hispanic | 5944 (5.93) | 4967 (5.84) | 977 (6.54) |  | 1506 (5.54) | 1274 (5.40) | 232 (6.40) |  |
| Non-Hispanic White | 29521 (65.29) | 25405 (65.25) | 4116 (65.51) |  | 8466 (68.98) | 7278 (68.91) | 1188 (69.45) |  |
| Non-Hispanic Black | 18034 (12.01) | 14833 (11.54) | 3201 (14.83) |  | 3622 (10.73) | 3057 (10.42) | 565 (12.68) |  |
| OtherRace-Including Multi-Racial | 6014 (7.14) | 5193 (7.17) | 821 (6.93) |  | 1368 (6.57) | 1183 (6.49) | 185 (7.06) |  |
| Smoking status, n(%) |  |  |  | **<0.001** |  |  |  | **<0.001** |
| Never | 24046 (53.51) | 21226 (54.26) | 2820 (48.76) |  | 9884 (52.17) | 8746 (53.11) | 1138 (46.20) |  |
| Former | 10992 (24.56) | 9479 (24.21) | 1513 (26.75) |  | 4810 (26.14) | 4131 (25.70) | 679 (28.87) |  |
| Now | 9167 (21.94) | 7785 (21.53) | 1382 (24.49) |  | 3866 (21.70) | 3269 (21.19) | 597 (24.93) |  |
| Education, n(%) |  |  |  | **0.013** |  |  |  | 0.415 |
| Less than high school | 34473 (33.38) | 29343 (33.11) | 5130 (34.99) |  | 5293 (17.94) | 4698 (18.13) | 595 (16.75) |  |
| High school or equivalent | 11557 (20.80) | 10050 (21.02) | 1507 (19.45) |  | 4328 (25.01) | 3762 (25.03) | 566 (24.92) |  |
| college or above | 21608 (45.82) | 18547 (45.87) | 3061 (45.56) |  | 8929 (57.04) | 7675 (56.84) | 1254 (58.33) |  |
| Alcohol use, n(%) |  |  |  | 0.067 |  |  |  | 0.085 |
| Mild drinking | 12270 (48.79) | 10653 (48.96) | 1617 (47.78) |  | 5645 (50.38) | 4906 (50.75) | 739 (48.15) |  |
| Moderate drinking | 8093 (33.85) | 6910 (33.50) | 1183 (36.00) |  | 3603 (33.00) | 3067 (32.51) | 536 (35.98) |  |
| Heavy drinking | 4596 (17.36) | 4026 (17.54) | 570 (16.23) |  | 2044 (16.62) | 1779 (16.74) | 265 (15.87) |  |
| Physical activity, n(%) |  |  |  | 0.366 |  |  |  | 0.106 |
| Low physical activity | 15677 (31.92) | 13490 (31.81) | 2187 (32.59) |  | 5663 (33.43) | 4896 (33.11) | 767 (35.42) |  |
| High physical activity | 30434 (68.08) | 25940 (68.19) | 4494 (67.41) |  | 9140 (66.57) | 7890 (66.89) | 1250 (64.58) |  |
| Hypertension, n(%) |  |  |  | **0.002** |  |  |  | **0.001** |
| No | 32029 (66.13) | 27799 (66.49) | 4230 (63.90) |  | 10698 (63.21) | 9386 (63.72) | 1312 (59.98) |  |
| Yes | 18572 (33.87) | 15934 (33.51) | 2638 (36.10) |  | 7807 (36.79) | 6704 (36.28) | 1103 (40.02) |  |
| Metabolic syndrome, n(%) |  |  |  | 0.287 |  |  |  | **0.038** |
| No | 18517 (77.59) | 15884 (77.76) | 2633 (76.58) |  | 12854 (74.64) | 11246 (75.00) | 1608 (72.41) |  |
| Yes | 4642 (22.41) | 3954 (22.24) | 688 (23.42) |  | 4443 (25.36) | 3798 (25.00) | 645 (27.59) |  |
| CKD, n(%) |  |  |  | 0.505 |  |  |  | 0.353 |
| No | 55582 (87.07) | 48026 (87.11) | 7556 (86.79) |  | 15071 (86.94) | 13113 (87.06) | 1958 (86.18) |  |
| Yes | 10170 (12.93) | 8774 (12.89) | 1396 (13.21) |  | 3287 (13.06) | 2856 (12.94) | 431 (13.82) |  |
| Cardiovascular diseases, n(%) |  |  |  | **<0.001** |  |  |  | **<0.001** |
| No | 38316 (91.21) | 33647 (91.78) | 4669 (87.56) |  | 16366 (91.24) | 14357 (91.87) | 2009 (87.29) |  |
| Yes | 5150 (8.79) | 4245 (8.22) | 905 (12.44) |  | 2106 (8.76) | 1714 (8.13) | 392 (12.71) |  |
| NAFLD, n(%) |  |  |  | **0.002** |  |  |  | **<0.001** |
| No | 12840 (70.42) | 11031 (71.06) | 1809 (66.61) |  | 7690 (66.21) | 6809 (67.23) | 881 (59.59) |  |
| Yes | 5013 (29.58) | 4249 (28.94) | 764 (33.39) |  | 4279 (33.79) | 3657 (32.77) | 622 (40.41) |  |

***Abbreviations:*** *SE, standard error; BMI, body mass index; PIR, poverty income ratio; CTI, C-reactive protein–triglyceride–glucose index; NAFLD, non-alcoholic fatty liver disease; CKD, chronic kidney disease.*

**Supplementary Table 2. Association between CTI and asthma risk in the metabolically unhealthy obese subgroup**

| Variables | Model1 | |  | Model2 | |  | Model3 | |
| --- | --- | --- | --- | --- | --- | --- | --- | --- |
|  | OR (95%CI) | *P-value* |  | OR (95%CI) | *P-value* |  | OR (95%CI) | *P-value* |
| CTI | 1.29 (1.15 ~ 1.45) | **<0.001** |  | 1.34 (1.12 ~ 1.61) | **0.002** |  | 1.29 (1.01 ~ 1.64) | **0.041** |

***Abbreviations:*** *OR, odds ratio; CI, confidence interval; CTI, C-reactive protein–triglyceride glucose index; NAFLD, non-alcoholic fatty liver disease; CKD, chronic kidney disease.*

***Model 1:*** *No adjustment was made for potential confounders.****Model 2:*** *Adjusted for age, PIR, sex, ethnicity, marital status, smoking status, educational level, amount of alcohol consumed, physical activity, BMI, and calorie intake on the first day.****Model 3:*** *Adjusted for age, PIR, sex, ethnicity, marital status, smoking status, educational level, amount of alcohol consumed, physical activity, calorie intake on the first day, BMI, hypertension, cardiovascular disease, CKD, NAFLD, and glycated hemoglobin..*

**Supplementary Table 3. Association between CTI and asthma risk in the never-smoker subgroup**

| Variables | Model1 | |  | Model2 | |  | Model3 | |
| --- | --- | --- | --- | --- | --- | --- | --- | --- |
|  | OR (95%CI) | *P-value* |  | OR (95%CI) | *P-value* |  | OR (95%CI) | *P-value* |
| CTI | 1.06 (0.98 ~ 1.15) | 0.136 |  | 1.10 (0.98 ~ 1.22) | 0.097 |  | 1.23 (1.04 ~ 1.47) | **0.020** |

***Abbreviations:*** *OR, odds ratio; CI, confidence interval; CTI, C-reactive protein–triglyceride glucose index; NAFLD, non-alcoholic fatty liver disease; CKD, chronic kidney disease.*

***Model 1:*** *No adjustment was made for potential confounders.****Model 2:*** *Adjusted for age, PIR, sex, ethnicity, marital status, smoking status, educational level, amount of alcohol consumed, physical activity, BMI, and calorie intake on the first day.****Model 3:*** *Adjusted for age, PIR, sex, ethnicity, marital status, smoking status, educational level, amount of alcohol consumed, physical activity, calorie intake on the first day, BMI, hypertension, cardiovascular disease, metabolic syndrome, CKD, NAFLD, and glycated hemoglobin.*
